# *Legionella longbeachae* infection in a persistent hand-wound after a gardening accident

**DOI:** 10.1099/jmmcr.0.004374

**Published:** 2014-12-01

**Authors:** Silja Mentula, Jaana Pentikäinen, Outi Perola, Eija Ruotsalainen

**Affiliations:** ^1^​ Bacteriology Unit, National Institute for Health and Welfare, P.O. Box 30, FI-00271 Helsinki, Finland; ^2^​ Eastern Finland Laboratory Centre Joint Authority Enterprise (ISLAB), Puijonlaaksontie 2, 70211 Kuopio, Finland; ^3^​ Kuopio University Hospital, Puijonlaaksontie 2, 70210 Kuopio, Finland

**Keywords:** extra-pulmonary, *Legionella*, *Legionella longbeachae*, soil, wound

## Abstract

**Introduction:**

Unlike other *Legionella* species, *Legionella longbeachae* has been associated with soil and potting composts instead of water systems, and it has caused pneumonia in gardeners.

**Case presentation:**

We report, to our knowledge, the first case of prolonged localized *L. longbeachae* infection in an accidental wound on the back of a hand caused by a broken flowerpot.

**Conclusion:**

Identification of *L. longbeachae* requires awareness and expertise, since commercial tests are most often specific for *L. pneumophila*.

## Introduction

In addition to respiratory symptoms, Legionnaires’ disease is associated with gastrointestinal and neurological symptoms, and can also involve the liver, kidneys and skin (Edelstein, 2011). *Legionella* can cause extra-pulmonary infections such as endocarditis and sternal-wound infections, also without clear respiratory symptoms, originating most often from contaminated tap water (Lowry *et al*., 1991; Leggieri *et al.*, 2012). While *Legionella pneumophila* sg 1 remains the predominant pathogenic strain, other *Legionella* species are emerging as significant pathogens. After decades of high incidence mostly in Australia and New Zealand (Li *et al*., 2002), *L. longbeachae* cases are increasing globally (Whiley & Bentham, 2011) and increasing numbers of *L. longbeachae* pneumonia cases have been reported, e.g. from Thailand and Scotland (Phares *et al*., 2007; Potts *et al*., 2013). *L. longbeachae* differs from other *Legionella* species by special risk factors and habitat; it has been connected to potting soil and potting composts instead of man-made water systems or other aquatic systems (Steele *et al*., 1990; Currie *et al*., 2014). Although numerous *Legionella* species have been isolated from soil, *L. longbeachae* is the only species for which soil has been reported as the source of infection (Currie *et al*., 2014; Cramp *et al*., 2010). Infection seems to be connected to gardening behaviour, rather than mere contact with potting soil. Although the precise mechanism of transmission is unknown, the risk of *L. longbeachae* infection is increased by lack of hand washing after gardening before eating or drinking, by a history of smoking, and by exposure to dripping water from flower pots (O’Connor *et al*., 2007).

## Case report

We report, to our knowledge, the first case of a localized infection by *L. longbeachae* in an accidental wound on the back of the hand from a broken flowerpot. This is the first extra-pulmonary case and the fifth reported case of *L. longbeachae* in Finland, the first case dating back to 1989.

An 80-year-old woman was admitted to the surgical emergency clinic with fever and infection of her right hand. She had a history of chronic atrial fibrillation, hypertension, osteoporosis and spinal stenosis, as well as polymyalgia rheumatica requiring continuous oral steroids. An infected cut on her right second finger from a dirty, wet, broken flowerpot 9 days earlier had not responded to intravenous (i.v.) cefuroxime initiated 2 days earlier.

On admission, she was febrile to 38.5°C. The wound over her right second interphalangeal joint was swollen; surrounding erythema extended almost to the elbow, but there was no evidence of abscess or crepitation. Blood analysis revealed an elevated C-reactive protein (CRP) of 54 mg l^−1^ and total leucocyte count of 9.5 E9 l^−1^. Therapy was begun with i.v. piperacillin/tazobactam, then continued with oral amoxicillin, for a total duration of 10 days. Signs of infection partially resolved and CRP dropped to 21, but mild pain and erythema on the back of the right hand persisted.

One month later, because of persistent pain, the right third and fourth metacarpophalangeal MCP joints were injected twice over 2 weeks with corticosteroids for presumed reactive arthritis. Because bacterial infection could not be completely excluded, a course of oral cefalexin was prescribed with the second injection.

The following week, the patient fell over, requiring suturing of a wound on her right elbow. Three days later she returned with increased swelling and redness of her right hand, but no fever. Although CRP was only 7 mg l^−1^ and leucocytes 10.1 E9 l^−1^, i.v. clindamycin was given for 5 days because of the significant history of infectious findings. The next day an ultrasound study revealed third MCP joint synovitis and flexor tendinitis but no abscess. The right third MCP joint was injected with corticosteroids for the third time with partial relief of the pain.

Within a week the patient returned to the hospital with worsening pain and inflammation of the hand. The CRP was only 7 mg l^−1^, but an ultrasound study revealed purulent arthritis and an abscess of the right third MCP joint. In addition, the wound on the right elbow had reopened with inflammation extending to the bone. A suspicious area of erythema and abnormal firmness was noted on the left thigh. Endocarditis was excluded. Antibiotic therapy was restarted with a combination of i.v. clindamycin and piperacillin/tazobactam. Both the elbow and hand wound were surgically debrided.

Altogether, three samples, one swab and two aspirates, were taken on three consecutive days from the hand abscess. Samples were cultured onto seven different agar plates routinely used for aerobic and anaerobic culture, and into anaerobic enrichment broth. Microscopic examination of one of the samples demonstrated a small amount of weakly stained Gram-negative rods without any special morphological features. Aerobic plates were incubated for 2 days, and anaerobic plates and enrichment broth for 5 days. All the cultures remained negative.

Because there was no clinical response after 5 days of this wide-spectrum antibiotic therapy, and because the only positive microbiological finding was a single positive Gram stain, the decision was made to forward the sample for bacterial PCR and DNA sequencing (Helsinki University Central Hospital laboratory HUSLAB, Finland). Based on the traumatic mechanism of injury, the suspicion of *Legionella* was aroused, and the samples were re-cultured onto Legionella-agar (BCYEα; Oxoid CM06655 and SR0110). After 4 days of incubation aerobically at +35 °C, abundant bacterial growth was detected on BCYEα agar. Colonies were small, greyish-white, with a faint blue tinge (catalase+, oxidase−). Gram staining revealed Gram-negative rods. Matrix-assisted laser desorption/ionization time-of-flight (MALDI-TOF) (Vitek MS; bioMérieux) identification was unsuccessful. Direct immunofluorescence assay (IFA) applying *L. pneumophila*-specific antiserum (Monofluo *Legionella pneumophila* IFA Test Kit; BioRad) remained negative. On the very same day, the bacterial PCR and sequencing result for the aspirate sample was reported as positive for *L. longbeachae* by HUSLAB. An isolate from BCYEα agar was sent to the National Institute for Health and Welfare (THL) for confirmation of preliminary *Legionella* identification. The species identification was confirmed by *mip* sequencing using the Public Health England protocol and database (http://www.hpa-bioinformatics.org.uk/cgi-bin/legionella/mip/mip_id.cgi). DNA was isolated by boiling growth from the BCYEα plate in 5 % Chelex.

Antibiotic therapy was switched to a four-week course of oral levofloxacin, and according to a follow-up phone call to the patient, resulted in the rapid resolution of all signs of infection.

## Discussion

Legionnaires’ disease can progress from respiratory infection to multisystem disease, or as in the present case, can remain as a localized extra-pulmonary infection for prolonged periods of time. Due in part to their rarity, *Legionella* infections are often underdiagnosed, especially in non-pulmonary sites. When diagnosis is delayed, recovery and rehabilitation from respiratory *L. longbeachae* infection can be slow even with targeted antibiotics (Wright *et al*., 2012). The diagnosis in our case was also delayed, but fortunately recovery was rapid once appropriate antibiotics were begun.

It is worth noting that *L. pneumophila*-specific urinary antigen and IFA tests do not detect *L. longbeachae*, and it was not in the MALDI-TOF database. Moreover, *Legionella* cultures, requiring specific growth media and conditions, may be negative for a week before growth appears. Serology and especially *Legionella* species PCR or sequencing can also be used in *Legionella* diagnostics. In the present case, *Legionella* was identified after active suspicion by an alert infectious disease specialist, but 2 months after the initial infection. When no other pathogens are found, the risk factors of a patient should be carefully identified, and the possibility of *Legionella* kept in mind. The specific risk factor for *L. longbeachae* infection is exposure to soil or potting compost while gardening.

## Abbreviations

CRP: C-reactive protein
IFA: immunofluorescence assay
i.v.: intravenous
MALDI-TOF: matrix-assisted laser desorption/ionization time-of-flight
MCP: metacarpophalangeal
